# Correction: Fici et al. An Updated Checklist of the Genus *Capparis* L. (Capparaceae) in Vietnam, including a New Species from Hon Tre Island. *Plants* 2022, *11*, 3402

**DOI:** 10.3390/plants12051055

**Published:** 2023-02-27

**Authors:** Silvio Fici, Leonid V. Averyanov, Danh Thuong Sy

**Affiliations:** 1 Department of Agricultural, Food and Forest Sciences, University of Palermo, Viale delle Scienze ed. 4, 90128 Palermo, Italy; 2 Komarov Botanical Institute of the Russian Academy of Science, Professor Popov Str. 2, 197376 St. Petersburg, Russia; 3 Faculty of Biology, TNU-University of Education, 20 Luong Ngoc Quyen, Thai Nguyen City 250000, Vietnam

The authors would like to report that the following corrections have been made to the original article [1]. 

In the Results section, the titles of the paragraphs were written in bold, several of the scientific names were italicized, and one reference number was corrected. In the Taxonomic Treatment section, the name of the new species was written in bold.

The authors apologize for any inconvenience caused and state that the scientific conclusions are unaffected. These corrections were approved by the Academic Editors. The original publication has also been updated.
